# Adaptive, diverse and de-centralized diagnostics are key to the future of outbreak response

**DOI:** 10.1186/s12915-020-00891-4

**Published:** 2020-10-28

**Authors:** Quinn Matthews, Severino Jefferson Ribeiro da Silva, Masoud Norouzi, Lindomar José Pena, Keith Pardee

**Affiliations:** 1 Leslie Dan Faculty of Pharmacy, University of Toronto, Toronto, Ontario Canada; 2 Department of Virology, Aggeu Magalhaes Institute (IAM), Oswaldo Cruz Foundation (Fiocruz), Recife, Pernambuco Brazil; 3 Department of Mechanical and Industrial Engineering, University of Toronto, Toronto, ON Canada

**Keywords:** COVID-19, Pandemic response, De-centralized diagnostics

## Abstract

The global spread of SARS-CoV-2 has shaken our health care and economic systems, prompting re-evaluation of long-held views on how best to deliver care. This is especially the case for our global diagnostic strategy. While current laboratory-based centralized RT-qPCR will continue to serve as a gold standard diagnostic into the foreseeable future, the shortcomings of our dependence on this method have been laid bare. It is now clear that a robust diagnostics pandemic response strategy, like any disaster planning, must include adaptive, diverse and de-centralized solutions. Here we look at how the COVID-19 pandemic, and previous outbreaks, have set the stage for a new innovative phase in diagnostics and a re-thinking of pandemic preparedness.

## Main

As World Health Organization Director General Dr. Tedros Adhanom Ghebreyesus said while highlighting the need for better coronavirus disease 2019 (COVID-19) testing, trying to contain an outbreak without adequate diagnostics is like attempting to “fight a fire blindfolded”. By identifying infected individuals and transmission hot-spots, diagnostic data enable scientists and governments to characterize an outbreak, allocate care and coordinate containment measures—without such data, mounting an effective pandemic response is nearly impossible. While the global response to the ongoing COVID-19 pandemic—which has infected millions and disrupted the global economy—has been admirable, the deployment of mass public health testing has been our most significant failure, making it clear that our capacity to respond to outbreaks relies too heavily on a single, rigid, centralized laboratory-based diagnostic modality. Thus, we now need to support the development of diverse, affordable and scalable diagnostic tools that can be taken out of the laboratory and into the field. By simply expanding our diagnostic reach into a more distributed network of testing, regardless of the context, we can bring tremendous near-term benefit and health security to the world.

## Current modality

**Fig. 1:**
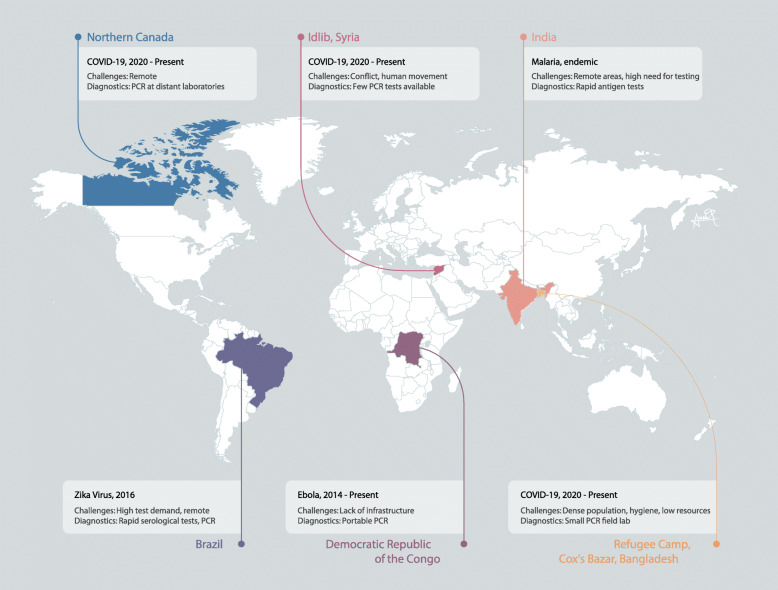
Global challenges to diagnostic access. A map of the world highlighting examples of the challenges faced in accessing diagnostics during public health crises, particularly in low-resource and remote locations

For decades, our national diagnostic strategies have been built around a laboratory-based testing model, where samples taken at the point-of-care (e.g. clinic, home, hospital) are delivered to a well-resourced centralized laboratory for processing and testing. Today, the molecular diagnostic workhorse in these settings is largely reverse transcription quantitative polymerase chain reaction (RT-qPCR), a technique which harnesses the enzymatic machinery of reverse transcription and DNA replication to amplify and detect specific target sequences (such as severe acute respiratory syndrome coronavirus 2 [SARS-CoV-2] RNA) in a patient sample [1]. While a remarkable tool with tremendous specificity and robustness, RT-qPCR is not a one-size-fits-all diagnostic; its requirement for trained laboratory personnel, expensive refrigerated reagents, cumbersome equipment and stable power supply mean that it is best suited for high-resource laboratories typically centred in urban areas. The result is a hub-and-spoke diagnostic network with gaps in rural and/or low-resource settings, and, as we have seen with COVID-19, insufficient capacity to respond to a surge in demand during public health crises. This challenge is exacerbated further during an outbreak situation, where the spatial epidemiology of an outbreak is rapidly changing and RT-qPCR lacks the necessary adaptive portability to respond (examples summarized in Fig. 1).

Our research groups observed these RT-qPCR challenges first-hand during the 2015–2016 Zika virus outbreak in Brazil, which infected over 200,000 people and left severe lasting pathologies in thousands of children [2]. As with COVID-19, centralized Zika virus diagnostics meant that just five references laboratories were tasked with testing the thousands of samples taken across the country. With each RT-qPCR test batch taking 2–3 h, lengthy backlogs arose, resulting in patients experiencing significant wait times. For rural or remote populations without direct access to these centralized facilities, samples had to be stored and transported within a cold chain for testing, further separating patients from timely results. These same challenges are often faced by those living in settings with endemic diseases, such as malaria.

Reliance on lab-based equipment and technical expertise also makes RT-qPCR difficult to deploy to the front lines; a significant downside during a rapidly evolving outbreak situation. This was the case during the 2013 West Africa Ebola outbreak—which claimed 11,323 lives—where it took over 80 days after the first deaths for reliable RT-qPCR testing to come on-line [3]. Because of infrastructure limitations, testing was initially carried out 6000 km away in a laboratory in Lyon, France. As the outbreak progressed, RT-qPCR field-testing laboratories closer to the front lines were established—however, challenges with testing capacity remained. The crisis left many wondering how many lives could have been saved if better de-centralized diagnostics were available earlier and, in many ways, was a catalyst for many of the field’s first attempts at de-centralizing molecular diagnostics [4].

Now, in the midst of COVID-19, we are facing a diagnostic crisis on a global scale. Millions of tests per day are needed, which, even if PCR reagent manufacturing is brought to scale, the simple logisitics of delivering such volume would be difficult without a significant investment in automation or, as we advocate, a shift toward de-centralized modalities. This is particularly challenging in remote areas and low-resource settings, where access to centralized infrastructure is lacking. For example, as of September 2020, testing of samples from Northern Canadian communities such as Yellowknife still required air transport to urban testing centres, in some cases, over a thousand kilometres away [5].

For the millions of refugees and displaced persons worldwide, access to diagnostic testing is even more tenuous. Often on the move or housed in refugee camps, these populations suffer from overcrowding (population densities can be double those of urban centres like Manhattan) and poor access to sanitation and clean water—a perfect storm for infectious disease spread. Because these camps are often under-resourced, their healthcare infrastructure is generally inadequate, making sufficient diagnostic testing capacity extremely difficult to implement. For example, refugee camps in Cox’s Bazar, Bangladesh—which house over 800,000 displaced Rohingya people—are served by just one testing laboratory, conferring a capacity of only 1000 tests per day—a far cry from the required capacity needed for COVID-19 surveillance [6].

Even well-resourced urban settings that are supported by established diagnostic networks are not immune from the chaos of an outbreak. Early in the outbreak, stringent criteria for testing eligibility were put in place in cities like Toronto due to a lack of surge capacity, resulting in unconfirmed patients and asymptomatic transmission unwittingly fueling disease spread [7]. Such bottlenecks have been aggravated by the worldwide reliance on RT-qPCR, which has led to supply chain disruption and forced governments to canvas universities and private laboratories for reagents that were in short supply. Taken together, these collective diagnostic shortfalls make a strong case for re-thinking our absolute dependence on RT-qPCR and a move toward augmenting this capacity with diverse and distributed tools that can adapt quickly to respond to urgent needs. As we discuss below, the development of these tools has begun and is hopefully just the first of a much larger and sustained push for diagnostic innovation.

## Going de-centralized

This new generation of diagnostic tools should be affordable to allow for broad distribution; user-friendly, to reduce the need for trained technicians; and portable, to expand the current diagnostic reach. These diagnostics will relieve pressure from centralized testing facilities and, importantly, help to stem the spread of infection by providing on-site results in near real-time, bringing testing capacity to hot spots (e.g. long-term care homes) and remote areas (e.g. refugee camps, rural communities) where sufficient diagnostic readiness is difficult to achieve. The availability of such affordable point-of care diagnostics will be key to prevent the second waves of disease and the reemergence of a given pathogen in disease-free areas.

Furthermore, implementing diverse testing methods will introduce new supply chains, mitigating risks associated with our global dependence on RT-qPCR reagents, consumables and instrumentation. These emerging diagnostics (summarized in Table 1) typically fall into three categories: antibody-based rapid antigen tests designed to detect SARS-CoV-2 antigens, protein-based serological assays designed to detect anti-SARS-CoV-2 antibodies or molecular diagnostics designed to detect viral RNA [1]. With each category serving different needs, all three have a place in a comprehensive diagnostic strategy.

**Table 1:** An overview of diagnostic platforms developed for the detection of SARS-CoV-2

Platform	Technology	Pros	Cons	Examples
**RT-qPCR**	DNA replication enzymes cycle through different temperatures to amplify specific SARS-CoV-2 sequences	• Well-established	• Requires centralized infrastructure	Thermo-Fisher TaqPath RT-qPCR COVID-19 kit
		• Gold standard for sensitivity and specificity		
			• Complex operation	
			• Slow (2–3 h)	
**Antigen assays**	Immobilized antigen-specific antibodies bind and detect SARS-CoV-2 antigens in patient fluid	• Portable	• Poor detection during early-stage infection	Abbott BinaxNOW COVID-19 Ag Card, SDBiosensor STANDARD Q COVID-19 Ag
		• Simple operation		
		• Affordable		
		• Ample sensitivity		
**Serological assays**	Immobilized viral proteins bind and detect anti – SARS-CoV-2 antibodies in patient fluid	• Portable	• Unable to detect early-stage infection	Cellex qSARS-CoV-2 IgG/IgM Rapid Test, Biolidics 2019-nCoV IgG/IgM Detection Kit
		• Simple operation		
		• Can detect past infection		
**CRISPR**	CRISPR gene-editing enzyme cleaves SARS-CoV-2 sequences, generating colour output	• Simple operation	• New technology not well established	SHERLOCK, DETECTR
		• Sensitive (20–200 aM)		
			• Not portable	
**Toehold switch**	Engineered gene circuits produce a colour upon detection of SARS-CoV-2 RNA	• Simple operation	• Still in development	Not yet approved for SARS-CoV-2
		• Low-cost		
		• Easy storage conditions		
**Isothermal amplification**	DNA replication enzymes active at a single temperature amplify SARS-CoV-2 sequences	• Portable	• May require specialized instrumentation	Abbott ID-NOW
		• Simple operation		
		• Sensitive, specific		
		• Quick (as fast as 5 min)	• Difficult to scale up	

Antibody-based antigen tests detect viral proteins in patient fluid in a manner similar to lateral flow pregnancy tests and have been deployed successfully for diagnosis of other infectious diseases like Malaria [1, 8]. Dependent only on an interaction between a SARS-CoV-2 antigen in a patient sample and an affixed antibody, these tests are rapid, portable, and inexpensive, providing results in 15–30 min and costing as little as $5 United States Dollars (USD). Though these tests lack the sensitivity of molecular diagnostics like RT-qPCR and are best suited for detection of virus during peak infection, they are poised to drastically improve distributed screening. Fifty million antigen tests per month are set to be manufactured by Abbott Laboratories starting in October 2020, and from this, and other sources, 120 million tests have been secured for low- and middle-income countries where RT-qPCR testing may be unattainable. The introduction of new testing capacity at this scale represents an exciting addition to the COVID-19 diagnostic landscape and will be especially valuable for distributed testing in the community.

Serological assays also resemble lateral flow pregnancy tests, where patient fluid passes over immobilized viral antigen and a colourimetric readout is produced if patient antibodies are detected. Though these assays are of limited value for diagnosis of active infections (antibodies may take days for a patient to generate and may persist long after infection), their portability and scalability make them promising decentralized tools for tracking individual and population-level virus exposure [1]. Such tools may be of considerable use as vaccines become available and countries need to make strategic use of precious vaccine stocks.

Molecular strategies include isothermal nucleic acid amplification-based diagnostics, which are a simpler and more portable alternative to conventional RT-qPCR amplification, and can be deployed to peripheral testing sites with far less infrastructure than conventional facilities [1]; toehold switch-based diagnostics, which use affordable and portable paper-based engineered gene circuits capable of detecting viral RNA; and, perhaps most excitingly, clustered regularly interspaced short palindromic repeats (CRISPR)-based assays, which harness CRISPR associated proteins programmed to recognize specific sites within the SARS-CoV-2 genome, generating a colour-based response [1]. CRISPR, which has been touted as one of the most impactful scientific discoveries of the past century, is now poised to make major contributions to diagnostics, with the specific high-sensitivity enzymatic reporter unlocking (SHERLOCK) and SARS-CoV-2 DNA endonuclease-targeted CRISPR trans reporter (DETECTR) platforms receiving landmark Food and Drug Administration (FDA) emergency usage authorizations (EUAs) for COVID-19 and exhibiting limits of detection similar to those of many RT-qPCR tests [9]. Collectively, these emerging molecular methods have the potential to enable a more distributed diagnostic network by complementing RT-qPCR and, importantly, their independence from the PCR supply brings new testing capacity.

Another implemented diagnostic tool worth highlighting is the Xpert SARS-CoV-2 platform from Cepheid, which is a semi-portable RT-PCR system that can be moved to peripheral testing sites at the front lines of an outbreak. This platform—which has been successfully deployed during recent Ebola outbreaks—has also received FDA EUA for SARS-CoV-2 and operates with a cartridge-based design, similar to a single-serve coffeemaker. While exciting, the widespread use of Cepheid is limited by equipment cost ($17,500 USD for a 4-module system) and low throughput, with a separate cartridge ($19.80 USD) needed for each test [10].

With the introduction of these novel diagnostics, it is a point of interest to imagine how the future of infectious disease diagnosis and screening will change. For formal clinical diagnostics, RT-qPCR—due to its high sensitivity, specificity and familiarity—will likely remain the gold standard but will be supplemented with different complementary options for large-scale screening and surveillance. For example, thanks to their portability and affordability, rapid antigen tests and CRISPR-based tests could be deployed in the field, such as in long-term care facilities or for routine screening in schools and other high-risk settings. This would allow central RT-qPCR testing capacity to be used exclusively to test symptomatic patients and to confirm positive results detected using rapid tests.

As these tests begin to enter the diagnostic market, only time can tell how they will fit into the changing diagnostic network. Supply chains need to be solidified, scale-up needs to be achieved, and tests need to be optimized and improved; all significant challenges during a global pandemic. Moreover, the continued development of new sensor technologies is critical and will pave the way to toward a more robust pandemic response with greater diagnostic sensitivity, specificity, speed and the ability to multiplex.

## A call to arms

While other areas of medicine have seen remarkable advancements over the last few decades, the diagnostic market has remained somewhat of an innovative doldrum. While the factors behind this are complex, the low profit margins of diagnostic reimbursement in healthcare systems have contributed in diminished investment in new diagnostic technologies. Now, having exposed the faults of our current approach, the COVID-19 pandemic gives us the opportunity to rethink and revitalize the diagnostic landscape. The human and economic cost of not doing so is all too clear.

Though a trying time, the lessons and new technologies that result from confronting COVID-19 will likely forever change how we think about diagnostics, and, more broadly, prepare for pandemics. Importantly, while developing and implementing COVID-19 diagnostics are certainly top-of-mind, our response to the pandemic is also an opportunity to re-think diagnostic infrastructure more broadly; by improving access to diagnosis of other diseases and infections, we can help extend the reach of health care out beyond the clinic, improve patient outcomes, and help push the world toward equitable access to healthcare for all.

## Data Availability

Not applicable
